# Bismuth–Titanium–Silicate–Oxide Glass Ceramics for Various Dielectric Applications

**DOI:** 10.3390/ma18245519

**Published:** 2025-12-08

**Authors:** Stanislav Slavov, Veselin Stankov

**Affiliations:** 1 Department of Mathematics, University of Chemical Technology and Metallurgy, 1797 Sofia, Bulgaria; 2 Department of Mechanics, University of Chemical Technology and Metallurgy, 1797 Sofia, Bulgaria; stankov_veselin@uctm.edu

**Keywords:** bismuth titanate, glass ceramics, dielectric characteristics

## Abstract

Ceramics based on bismuth titanate with added SiO_2_ and Nd_2_O_3_ were synthesized from the Bi_2_O_3_–TiO_2_–SiO_2_–Nd_2_O_3_ system through rapid melt quenching followed by controlled cooling. By adjusting the initial compositions and applying heat treatments between 1450 °C and 1100 °C, either homogeneous crystalline products or multiphase glass–ceramics were obtained. The identified crystalline phases included Bi_12_TiO_20_ and Bi_4_Ti_3_O_12_, coexisting with amorphous networks enriched in silicon, bismuth, titanium, and aluminum oxides. In previous investigations, the materials were characterized using X-ray diffraction, scanning electron microscopy, and Fourier-transform infrared spectroscopy, which collectively confirmed the presence of both ordered and disordered structural domains within the bulk samples. Electrical properties were evaluated through measurements of conductivity (4 × 10^−9^ S/m to 30 S/m), dielectric constant (real part from 10 to 5 × 10^3^ and imaginary part from 5 to 5 × 10^4^), and dielectric loss (0.02 to ~100) over the frequency range 1 Hz–1 MHz. These results provide a foundation for rational control of phase evolution in this quaternary oxide system and highlight strategies for tailoring the functional properties of glass–ceramic materials for dielectric applications. The aim of the present study is to investigate the relationship between phase composition, structural features, and dielectric behavior in cast Bi–Ti–Si–Nd glass–ceramics. Particular attention is given to the influence of the amorphous network containing SiO_2_ as a traditional glass former, as well as the formation of amorphous crosslinking Si–O–Ti structures acting as non-traditional glass formers.

## 1. Introduction

Recent investigations highlight the pivotal role of Bi_4_Ti_3_O_12_ (BIT) in the Bi_2_O_3_–TiO_2_ system, emphasizing the strong interdependence between processing conditions, microstructural features, and the resulting dielectric and ferroelectric performance. Aurivillius-type layered oxides continue to attract considerable interest as candidates for high-temperature dielectrics, environmentally friendly piezoelectrics, and advanced thin-film applications, building upon the long-established foundation of their use in electronic and piezoelectric devices [1,2,3,4]. Current research maintains a strong focus on microstructural engineering through dopant incorporation and synthesis design, approaches already outlined in earlier surveys of ferroelectric ceramics and lead-free materials [5,6] and also processing–property correlations for both bulk and thin-film forms [7,8,9]. At the microstructural level, studies on BIT ceramics demonstrate that grain size and domain-wall arrangements (notably 180° and 90° boundaries) exert significant influence on hysteresis behavior and polarization switching, even when defect dipoles are present. These findings provide valuable guidelines for linking sintering strategies and dopant selection with targeted dielectric and polarization outcomes [10]. Complementary efforts on doped BIT compositions reveal that substitutions at both the A- and B-sites—particularly with rare-earth ions—enhance the piezoelectric coefficient d_33_ while preserving the Curie temperature, offering practical approaches to performance optimization without resorting to lead-based systems [11,12,13,14,15]. In addition, co-doping strategies that mitigate oxygen vacancies improve insulation resistance and ensure thermal reliability, reinforcing the suitability of BIT ceramics for demanding high-temperature applications [16]. Thermodynamic and kinetic analyses have recently refined the understanding of phase development within the Bi_2_O_3_–TiO_2_ binary system. These studies define stability ranges and transformation pathways for critical phases such as Bi_12_TiO_20_, pyrochlore structures, and BIT itself, thereby enabling more accurate sintering protocols and melt-quenching processes that yield dense ceramics with reproducible dielectric characteristics [14]. Such advancements build upon the established phase diagram data, reducing uncertainties in processing–property correlations for both bulk and thin-film forms [7,8]. Two major reviews have further positioned Aurivillius phases within the context of lead-free ferroelectric research. One consolidates progress in compositional and structural tuning across the material family, while the other explores the design flexibility of layered ferroelectrics, focusing on key variables such as layer number (*n*), site-specific chemistry, and domain engineering. These factors are directly tied to critical material parameters, including remanent polarization (P_r_), coercive field (E_c), piezoelectric coefficient (d_33_), and thermal endurance [9,15]. Collectively, recent progress—ranging from doping strategies and domain tailoring to phase stability analysis—supports a converging view: BIT and related Aurivillius oxides achieve optimal performance when chemistry, microstructure, and fabrication techniques are carefully integrated to satisfy modern requirements for reliability, efficiency, and environmental responsibility [1,2,3,4,5,6,10,11,12,13,14,15,16,17].

## 2. Materials and Methods

In previous work [18], ceramic and glass–ceramic polyphase materials were synthesized by melt annealing and characterized by X-ray diffraction and infrared spectroscopy. In the present study, mixtures of Bi_2_O_3_, TiO_2_, SiO_2_, and Nd_2_O_3_ powders were processed at temperatures between 1450 °C and 1100 °C, depending on composition. The synthesis procedure comprised a 15 min homogenization of the oxide mixture followed by melting in aluminum crucibles placed in a SiC tube furnace (KTM-GSL1700X, Xiamen Tmax Battery Equipments Limited, Fujian, China). The melts were then cooled to room temperature in graphite crucibles (Table 1). Corundum crucibles (Al_2_O_3_) were used for all melting experiments. To minimize aluminum incorporation, the maximum heating time for each batch was limited to 20 min. Microstructural analysis was carried out using SEM/EDS (Carl Zeiss EVO 10, Carl Zeiss, Oberkochen, Germany). Structural characterization was further performed using FTIR spectroscopy (Thermo Nicolet Avatar 370, Thermo Nicolet Corporation, Madison, WI, USA) and Raman spectroscopy (Renishaw inVia Qontor, Renishaw Plc, Renishaw, UK).

The cooling rate was approximately 100 K·min^−1^, achieved by allowing the melts to freely cool to room temperature. The texture of the materials was previously established in our earlier work [18] through comparison of the measured diffraction intensities with the characteristic reflections of the Bi_4_Ti_3_O_12_ and Bi_12_TiO_20_ phases. Sample dimensions were determined from three consecutive measurements and averaged, with a measurement uncertainty of ±2 µm. The specific sample sizes are listed in Table 2.

This study [18] provides an in-depth examination of the infrared spectra (400–1200 cm^−1^) of the investigated compositions, where the observed vibration bands correspond both to crystalline phases identified by XRD and to characteristic structural units. These include the following: (1) symmetric vibrations of Ti–O octahedra, (2) deformation modes of the TiO_6_ octahedron, (3) three-dimensional SiO_4_ units with four terminal oxygen atoms, (4) antisymmetric stretching of Si–O–Al and Si–O–Si frameworks, and (5) stretching vibrations of Al–O bonds in tetrahedral configurations [19].

Water absorption and Archimedes density were determined following the general principles of ASTM C373 (Table 3 and Table 4). The procedure consisted of four stages: drying, saturation, immersion weighing and calculation of the relevant parameters.

The experimental ρ_a_ values range between 4.99 and 6.12 g·cm^−3^, which is fully consistent with the behavior of Bi_2_O_3_-rich glass and glass–ceramic systems reported in the literature (typically 4.6–7.8 g·cm^−3^, depending on composition and crystallization degree). The theoretical densities ρ_mix, calculated from the nominal oxide compositions (including the Al_2_O_3_ incorporated from the crucible), fall within the interval 4.32–5.69 g·cm^−3^.

In the current work, the FTIR (FTIR, Thermo Nicolet Avatar 370) and the Raman (Renishaw inVia Qontor, 532 nm/785 nm) spectra of the compositions were further studied, as well as underwent mathematical processing of the results by (1) correction of the baseline (Asymmetric Least Squares), (2) smoothing (Savitzky–Golay), (3) normalization by area, and (4) detection of peaks with prominence by calculating center and FWHM (from half-high). The electrical characterization was performed using a Zahner IM6 impedance meter (Zahner Elektrik, Kronach, Germany). A two-point holder method was used, with silver electrodes deposited on the surfaces of the samples. The impedance modulus and phase angle were recorded as functions of frequency in the range 1 Hz–1 MHz at room temperature. Based on the dimensions of each sample, the dielectric constants (real and imaginary parts), dielectric losses, and electrical conductivity were calculated.

## 3. Results

**Figure 1:**
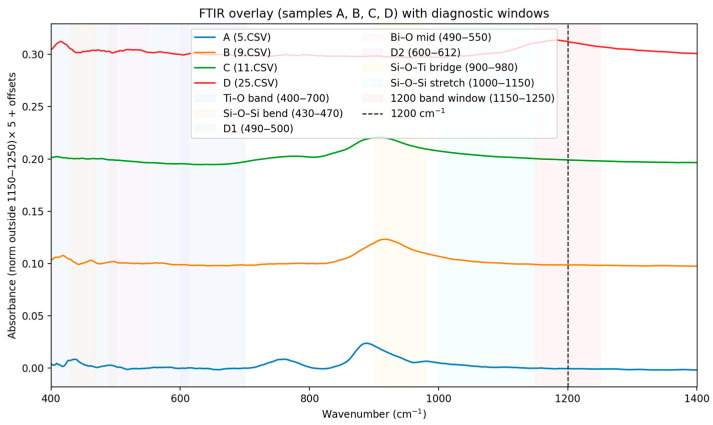
FTIR spectra of samples A, B, C and D (smoothing (Savitzky–Golay) and normalization by area).

In FTIR spectra (Figure 1) were baseline-corrected, lightly smoothed, and area-normalized (400–1200 cm^−1^). Diagnostic windows (Si–O–Si, Si–O–Ti, Ti–O, Bi–O) were integrated to compute indices and cosine similarity against IR fingerprints for Bi_4_Ti_3_O_12_ (Aurivillius) and Bi_12_TiO_20_, (Sillenite). Samples A and B: the higher Ti–O band index and the lower Bi–O signature in the IR are consistent with the Raman-based Aurivillius distribution (Bi_4_Ti_3_O_12_). Samples C and D: the increased Bi–O windows and the relatively weaker Ti–O band are consistent with the Sillenite character established by the Raman distribution (Bi_12_TiO_20_) [20,21,22]. Differences in selection rules (IR vs. Raman) may change the relative intensities, but the phase trend is consistent with both methods. Additional summarizing of Voigt-fit [23] parameters and PCA [24] clustering results for four Raman spectra Si peak (~520.7 cm^−1^) is stable across all samples. Ti (447 cm^−1^ and 612 cm^−1^) peaks show minor shifts indicating differences in strain and crystallinity [25,26,27,28]. -Sample C exhibits broader Ti peaks—suggesting higher defect density or dopant effects. -PCA clustering separates sample C and sample D from samples A and B, confirming compositional or structural variation. The structural variations are often determined by the appearance of glassy crosslinking, characteristic of silicate glasses. In this case, these are the identified D_2_ (~606 cm^−1^) and the ring nature of D_1_/D_2_ in silicate Si-O-Si bridge chains [29,30]. The strong absorption at ~1200 cm^−1^ is assigned to the longitudinal optical (LO) component of the asymmetric stretching mode νas (Si–O–Si) in a silicate network. Its high frequency and intensity indicate a highly polymerized framework (predominantly Q^4^ units), while the corresponding transverse optical (TO) component typically lies around 1060–1100 cm^−1^. Because this band reflects overall network connectivity rather than specific Bi–O or Ti–O phase fingerprints, we treat it as a reference feature and exclude the 1150–1250 cm^−1^ window from normalization/PCA to avoid biasing phase discrimination. Phase-sensitive comparisons are made primarily within 400–1100 cm^−1^ (Ti–O, Bi–O, and Si–O–M linkages).

**Figure 2:**
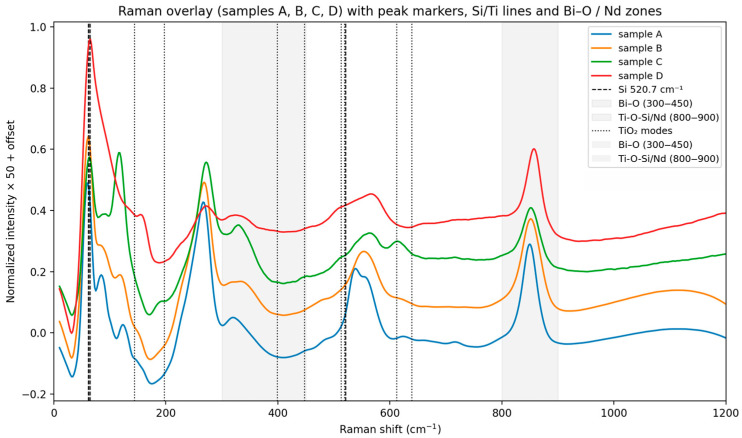
Raman spectra of samples A, B, C and D, (smoothing (Savitzky–Golay) and normalization by area).

At the same time, in the analysis of the Raman spectra (Figure 2), bands around 935–960 cm^−1^ are observed which are associated with localized stretching of Si–O–Ti in silicon titanates [25]. The characteristic band at about 960 cm^−1^ can be generally interpreted as typical of Si–O–Ti or Si–O influenced by Ti [26]. Additionally, the observed T–O–T bands at 480–520 cm^−1^ correspond to the characteristic Si–O–Al/Si–O–Si vibrational region [27,28,29,30,31,32].

**Figure 3:**
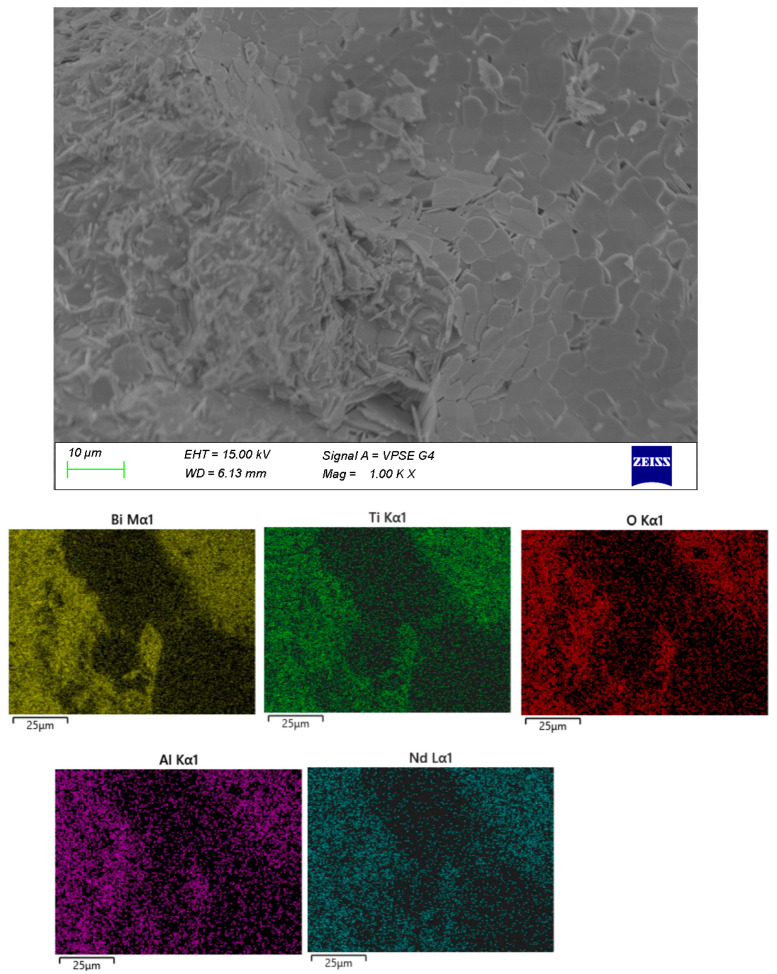
SEM and EDX of sample A.

**Figure 4:**
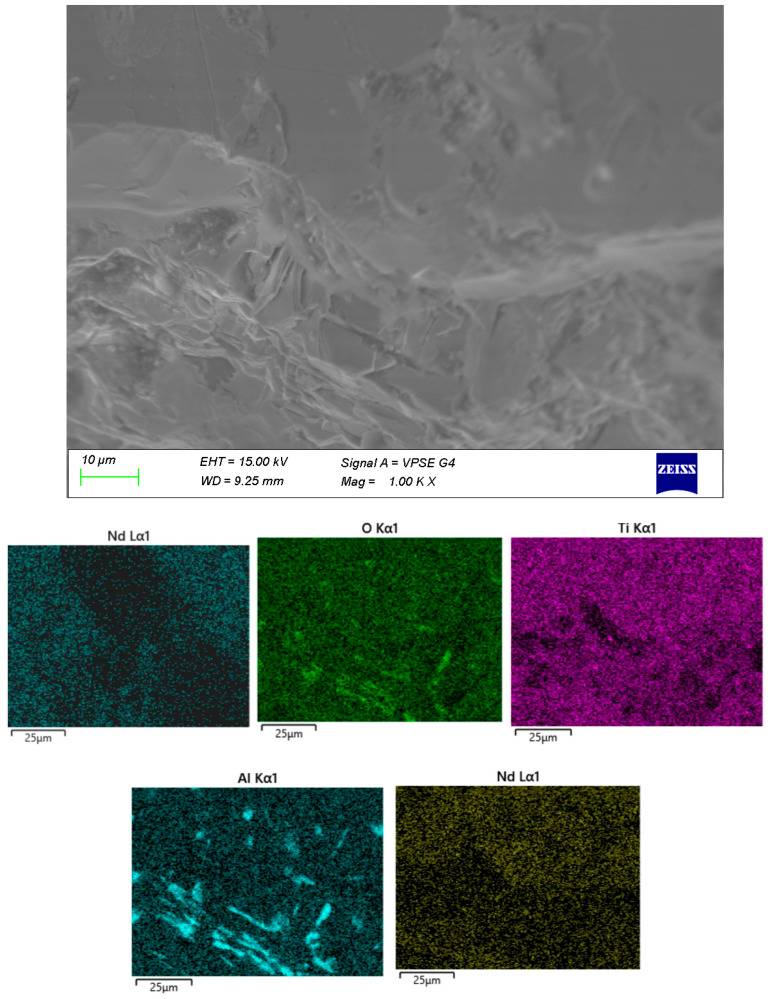
SEM and EDX of sample B.

**Figure 5:**
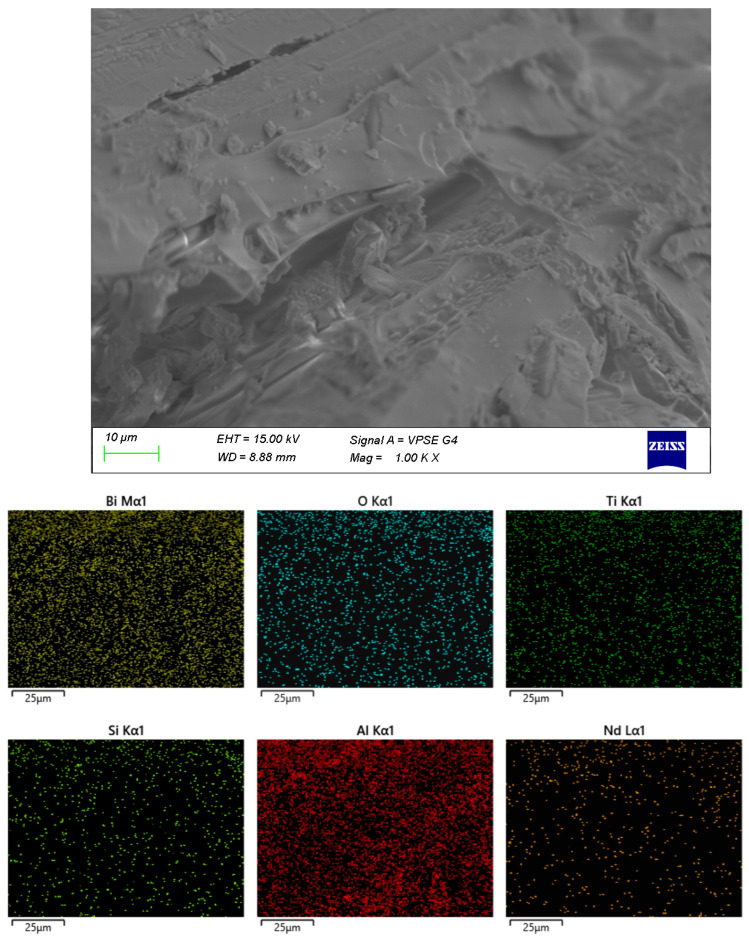
SEM and EDX of sample C.

**Figure 6:**
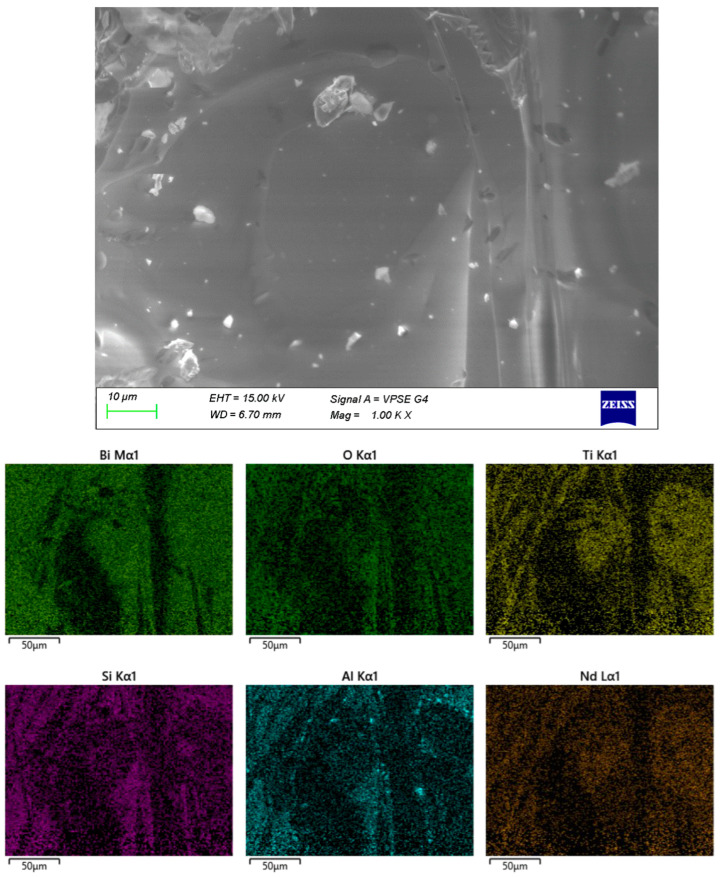
SEM and EDX of sample D.

The surface microstructure of the samples was examined by SEM, and the elemental distribution was verified by EDX (Figure 3, Figure 4, Figure 5 and Figure 6). In sample A (Figure 3), a uniform distribution of the constituent elements and a minimal aluminum content are clearly observed. The microstructure is dominated by crystalline formations primarily composed of Bi and Ti, while the presence of an amorphous matrix is scarcely discernible.

In sample B (Figure 4), the crystalline formations are embedded within a distinct amorphous matrix. The distribution of Al within the volume is highly irregular, most likely resulting from contact with the crucible during melting.

Similarly to sample B, sample C exhibits crystalline formations embedded in an amorphous matrix. A preferred orientation of the crystals is observed, and the distribution of Al within the volume is relatively heterogeneous.

Sample D contains the highest proportion of amorphous matrix, with only isolated crystalline inclusions. The distribution of Al within the volume is relatively heterogeneous, whereas Bi and Ti are concentrated in distinct localized regions.

The remaining samples (Figure 4, Figure 5 and Figure 6) exhibit varying distributions of crystalline inclusions within their glassy matrices, as confirmed by the XRD diffractograms. The ingress of Al influences glass formation by generating Al–O–Si bridge bonds. According to the EDX analysis, the Al contents are as follows: sample A—1.3 mol%, sample B—2.98 mol%, sample C—16.22 mol%, and sample D—7.84 mol%. Notably, at lower synthesis temperatures (sample A), the amount of incorporated Al is minimal.

**Figure 7:**
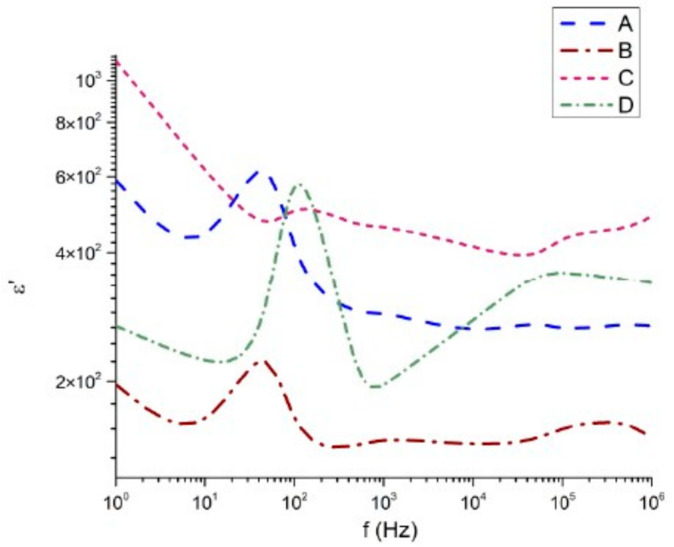
Real part of the dielectric permittivity as a function of the frequency.

**Figure 8:**
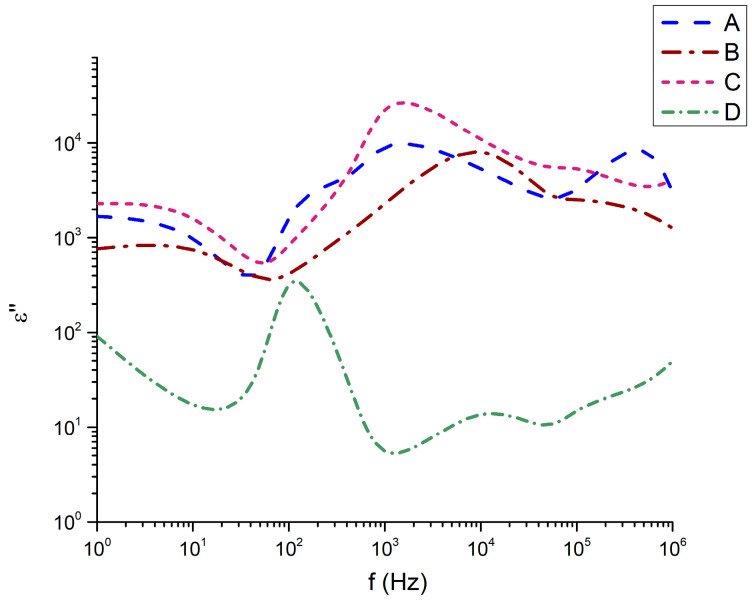
Imaginary part of the dielectric permittivity as a function of the frequency.

**Figure 9:**
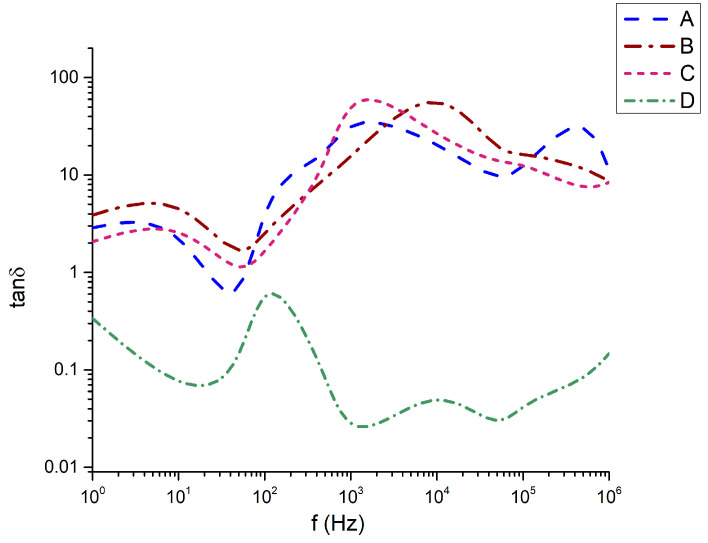
Dielectric losses as a function of the frequency.

**Figure 10:**
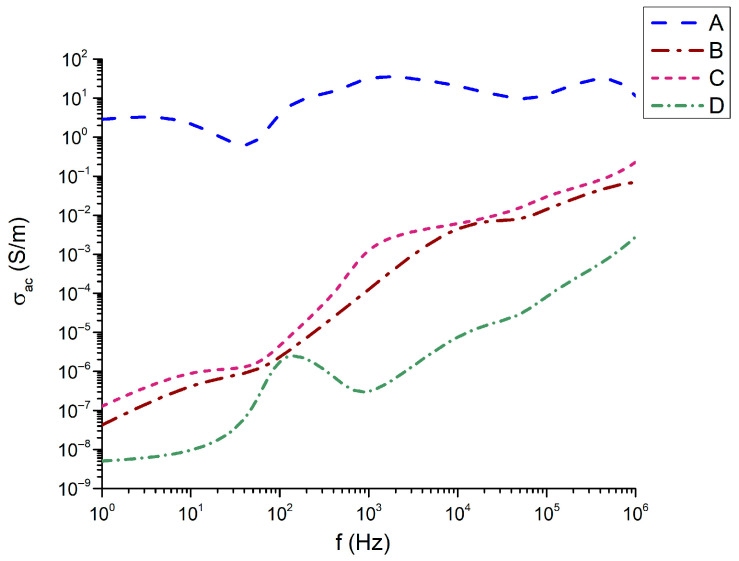
Conductivity as a function of the frequency.

The dielectric characteristics of the samples were measured in the frequency range from 1 Hz to 1 MHz. The dielectric permittivity (real and imaginary components), dielectric loss angle, and electrical conductivity are presented in Figure 7, Figure 8, Figure 9 and Figure 10, respectively. The real part of the dielectric permittivity of the studied samples 45Bi_2_O_3_-50TiO_2_-5Nd_2_O_3_ (A), 36Bi_2_O_3_-60TiO_2_-4Nd_2_O_3_ (B), 33Bi_2_O_3_-60TiO_2_-3SiO_2_-4Nd_2_O_3_ (C), and 32Bi_2_O_3_-38TiO_2_-22SiO_2_-8Nd_2_O_3_ (D), shown in Figure 7, remains within the range from 10 to 10^3^. There is a general tendency for the dielectric permittivity to decrease with increasing frequency. The composition 32Bi_2_O_3_-38TiO_2_-22SiO_2_-8Nd_2_O_3_ (D) is impressive, in which from 10^3^ Hz to 10^5^ Hz an increase of about 2 × 10^2^ and a local maximum is observed. Similar maximums of the permittivity at high frequencies are observed in the compositions 33Bi_2_O_3_-60TiO_2_-3SiO_2_-4Nd_2_O_3_ (C) and 36Bi_2_O_3_-60TiO_2_-4Nd_2_O_3_ (B), in which the increase begins after about 10^5^ Hz and is no more than 10^2^ for (C) and 0.2 for (B). At low frequencies, the samples without SiO_2_ content 45Bi_2_O_3_-50TiO_2_-5Nd_2_O_3_ (A) and 36Bi_2_O_3_-60TiO_2_-4Nd_2_O_3_ (B) have maxima at about 30 Hz with corresponding values of the dielectric constant of 6 × 10^2^ and 2.2 × 10^2^. In the case of SiO_2_ containing compositions 33Bi_2_O_3_-60TiO_2_-3SiO_2_-4Nd_2_O_3_ (C) and 32Bi_2_O_3_-38TiO_2_-22SiO_2_-8Nd_2_O_3_ (D), this maximum is slightly shifted to frequencies 1.1 × 10^2^ and values close to 6 × 10^2^.

It is noteworthy that sample 32Bi_2_O_3_-38TiO_2_-22SiO_2_-8Nd_2_O_3_ (D), which has the highest SiO_2_ content, demonstrates the highest instability in terms of frequency variation at the expense of TiO_2_. A similar strong sensitivity to the frequency of this sample effect is also observed in the imaginary part of the dielectric permittivity (Figure 8). While compositions 45Bi_2_O_3_-50TiO_2_-5Nd_2_O_3_ (A), 36Bi_2_O_3_-60TiO_2_-4Nd_2_O_3_ (B), and 33Bi_2_O_3_-60TiO_2_-3SiO_2_-4Nd_2_O_3_ (C) have almost similar behavior of the imaginary part of the dielectric permittivity, especially well expressed at low frequencies from 1 Hz to 10^3^ Hz, sample 32Bi_2_O_3_-38TiO_2_-22SiO_2_-8Nd_2_O_3_ (D) has completely different behavior.

This behavior directly corresponds to the dielectric losses (Figure 9), where it is also seen that while 45Bi_2_O_3_-50TiO_2_-5Nd_2_O_3_ (A), 36Bi_2_O_3_-60TiO_2_-4Nd_2_O_3_ (B), and 33Bi_2_O_3_-60TiO_2_-3SiO_2_-4Nd_2_O_3_ (C) have dielectric loss angles from 1 to 100, only sample 32Bi_2_O_3_-38TiO_2_-22SiO_2_-8Nd_2_O_3_ (D) demonstrates losses below 1 (from 0.02 to about 0.12).

It is interesting to note in Figure 10 that in the frequency dependence of the conductivity with the highest conductivity from about 1 to 10 is the composition 45Bi_2_O_3_-50TiO_2_-5Nd_2_O_3_ (A); the compositions with identical Nd_2_O_3_ content have identical frequency dependence and very close values of conductivity. Here again, the exception is the composition 32Bi_2_O_3_-38TiO_2_-22SiO_2_-8Nd_2_O_3_ (D), for which the conductivity remains the lowest with values from 4 × 10^−8^ S/m to 1.1 × 10^−3^ S/m.

With these results of the dielectric properties, it is imperative to pay attention not only to the crystalline phases present in the compositions, but also to the number of oxides included in the bismuth titanate, such as SiO_2_ and Nd_2_O_3_. In this regard, it is necessary to describe the influence of amorphous structural groups and phase structures based on Si-O-Si, Si-O-Ti, Ti-O and TiO_6_ octahedra.

**Figure 11:**
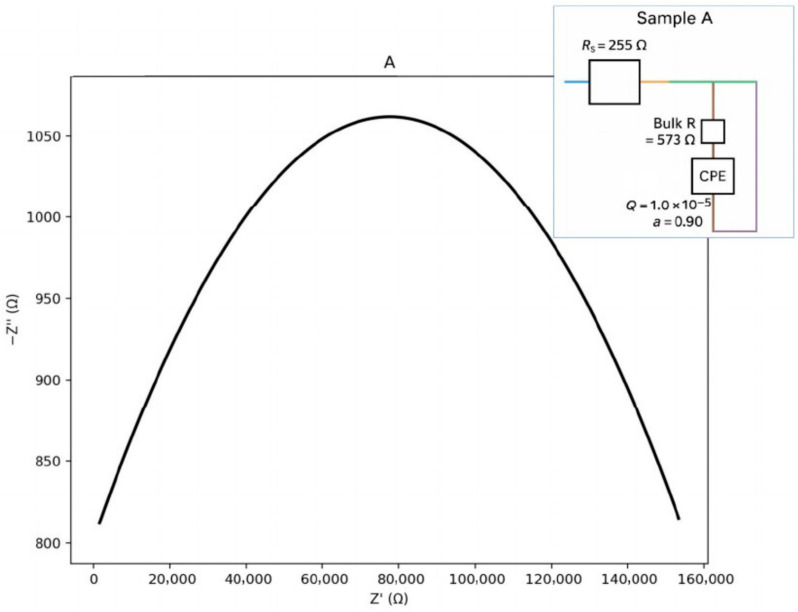
Nyquist plot with equivalent circuit for sample A.

**Figure 12:**
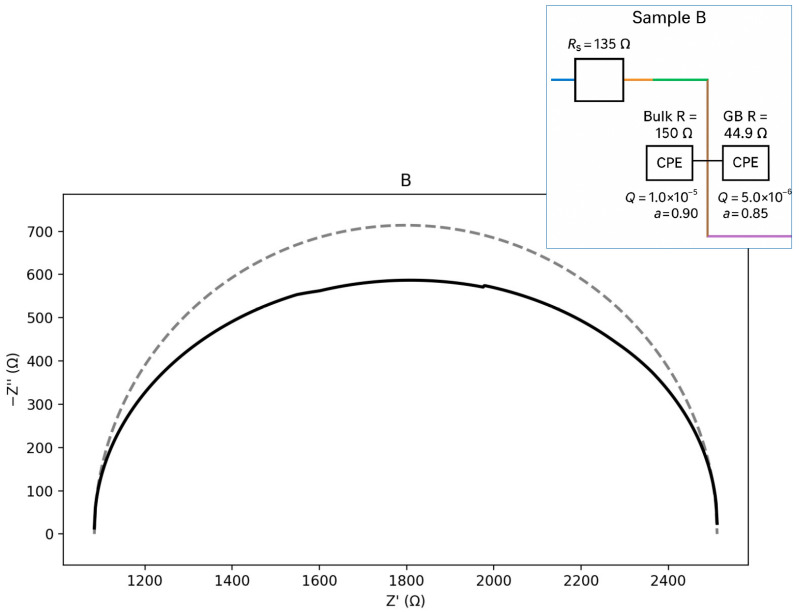
Nyquist plot with equivalent circuit for sample B.

**Figure 13:**
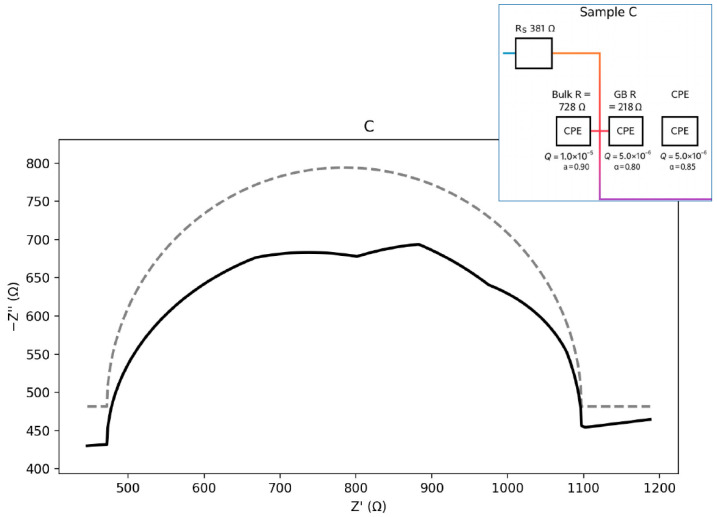
Nyquist plot with equivalent circuit for sample C.

**Figure 14:**
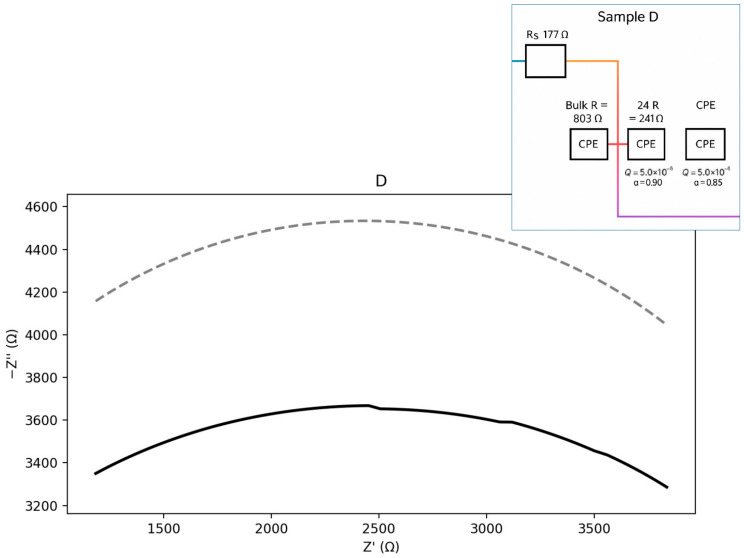
Nyquist plot with equivalent circuit for sample D.

The plots in Figure 11, Figure 12, Figure 13 and Figure 14 illustrate the dependence of the imaginary part of the dielectric constant on its real part over the studied frequency range. For the calculation of the equivalent circuits, the Constant Phase Element (CPE) exponent was applied according to the methodology described in [33,34,35,36,37]. Based on the proposed equivalent circuit models, concise conclusions were drawn for samples A–D, as summarized below:

$$Z_{CPE}\;=\;1/\left(Q{\left(j\omega \right)}^{a}\right),0\leq a\leq 1$$

$$Z_{R∥CPE}={\;(1/R+Q{\left(j\omega \right)}^{a})}^{-1}$$

$$\omega_{max}={\left(QR\right)}^{-1/a},f_{max}=\omega_{max}/\left(2\pi \right)$$

$$C_{eff}=Q^{1/a}R^{(1/a)-1}$$

For sample A (Figure 11), a single dominant bulk arc with low dispersion (a_b ≈ 0.90) is observed. This indicates a weak grain-boundary (GB) contribution with low-frequency features. The corresponding equivalent circuit can be represented as an Rs–(Rb ∥ CPEb) type. The calculated parameters are presented below:

Seeds: Rs ≈ 255.4 Ω; Rb ≈ 572.8 Ω; Qb = 1.0 × 10^−5^; a_b = 0.90.

$$Z_{b}={\left(1/R_{b}+Q_{b}{\left(j\omega \right)}^{0.90}\right)}^{-1}$$

$$\omega_{max},b={\left(Q_{b}R_{b}\right)}^{-1/0.90}$$

For sample B (Figure 12), the calculations indicate the presence of two-time constants, with the bulk response being nearly ideal. In this sample, the grain-boundary (GB) contribution is more strongly depressed, reflecting increased heterogeneity. The equivalent circuit can be represented as an Rs–(Rb ∥ CPEb)–(Rgb ∥ CPEgb) type. The calculated parameters are presented below:

Seeds: Rs ≈ 134.7 Ω; Bulk: Rb ≈ 149.8 Ω, Qb = 1.0 × 10^−5^, a_b = 0.90; GB: Rgb≈44.9 Ω,

Qgb = 5.0 × 10^−6^, a_gb = 0.85.

$$Z_{b}={\;(1/R_{b}+Q_{b}{\left(j\omega \right)}^{0.90})}^{-1}$$

$$Z_{gb}={\left(1/R_{gb}+Q_{gb}{\left(j\omega \right)}^{0.85}\right)}^{-1}$$

$$\omega_{max},b={\;(Q_{b}R_{b})}^{-1/0.90};\omega_{max},gb={\;(Q_{gb}R_{gb})}^{-1/0.85}$$

Sample C (Figure 13) exhibits higher resistances compared with sample B, along with a clearly depressed GB arc, indicating stronger barrier effects and increased heterogeneity. The corresponding equivalent circuit is of the Rs–(Rb ∥ CPEb)–(Rgb ∥ CPEgb) type. The calculated parameters are presented below:

Seeds: Rs ≈ 381.1 Ω; Bulk: Rb ≈ 727.8 Ω, Qb = 1.0 × 10^−5^, a_b = 0.90; GB: Rgb ≈ 218.3 Ω,

Qgb = 5.0 × 10^−6^, a_gb = 0.85.

$$Z_{b}={\left(1/R_{b}+Q_{b}{\left(j\omega \right)}^{0.90}\right)}^{-1}$$

$$Z_{gb}={\left(1/R_{gb}+Q_{gb}{\left(j\omega \right)}^{0.85}\right)}^{-1}$$

Sample D (Figure 14) exhibits the highest bulk resistance and a well-pronounced GB arc. The grain-boundary/interfacial contributions are therefore significant. The corresponding equivalent circuit can be represented as an Rs–(Rb ∥ CPEb)–(Rgb ∥ CPEgb) type. The calculated parameters are presented below:

Seeds: Rs ≈ 176.9 Ω; Bulk: Rb ≈ 803.0 Ω, Qb = 1.0 × 10^−5^, a_b = 0.90; GB: Rgb ≈ 240.9 Ω,

Qgb = 5.0 × 10^−6^, a_gb = 0.85.

$$Z_{b}={\;(1/R_{b}+Q_{b}{\left(j\omega \right)}^{0.90})}^{-1}$$

$$Z_{gb\;}={\left(1/R_{gb}+Q_{gb}{\left(j\omega \right)}^{0.85}\right)}^{-1}$$

For all samples except A, the measured ρ_a_ values exceed the calculated ρ_mix. This deviation becomes even clearer after correcting for residual porosity. The porosity-corrected densities ρ_true = 5.04–6.37 g·cm^−3^ approach or exceed densities typical for crystalline bismuth titanate phases such as Bi_2_Ti_4_O_11_ and Bi_4_Ti_3_O_12_, which are known to reach ρ ≈ 6.0–7.5 g·cm^−3^ in dense ceramic form. These observations strongly indicate that the glasses underwent substantive crystallization into high-density Bi–Ti–(Al) oxide phases during processing, consistent with previous structural analyses of similar systems.

**Figure 15:**
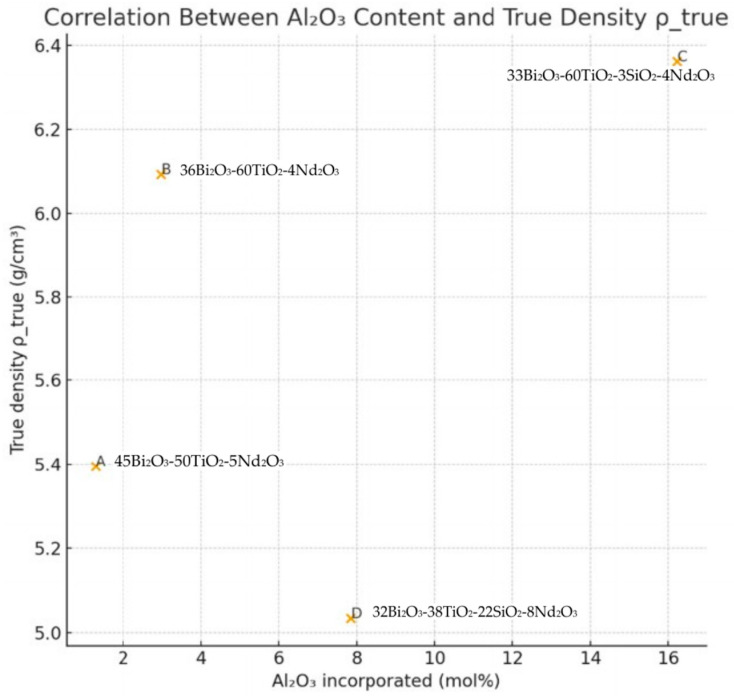
Relationship between Al_2_O_3_ content and ρ_true.

An additional factor influencing the density evolution is the incorporation of Al_2_O_3_ from the alumina crucible, quantified by EDS as 1.30, 2.98, 16.22 and 7.84 mol% for samples A–D, respectively. Figure 15 shows the relationship between Al_2_O_3_ content and ρ_true. Although Al_2_O_3_ itself has a lower intrinsic density (≈3.9–4.0 g·cm^−3^) compared to Bi_2_O_3_ and Nd_2_O_3_, the samples with higher Al incorporation (particularly sample C) exhibit higher ρ_true.

**Figure 16:**
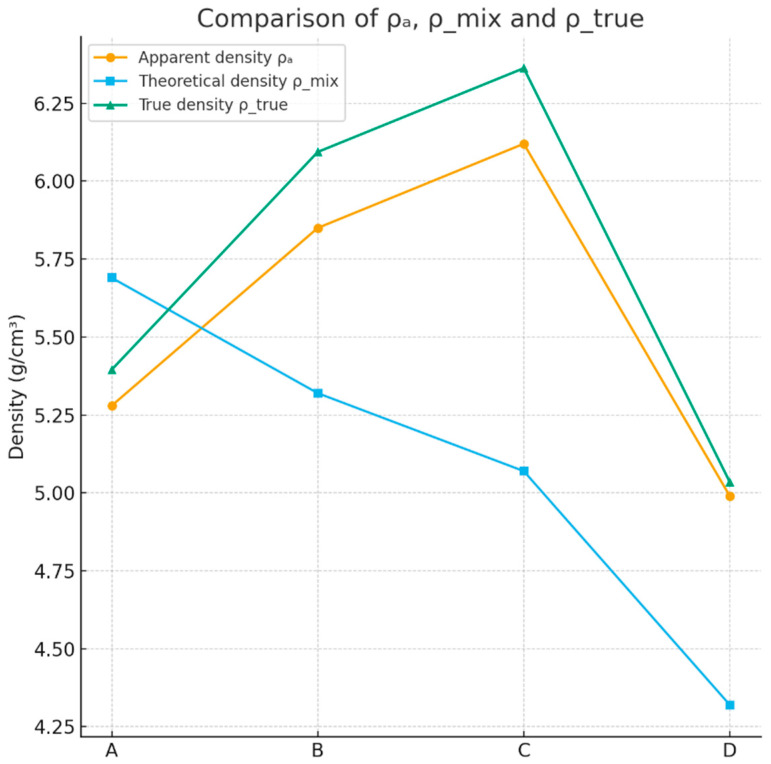
Comparative density analysis—combining measured ρ_a_, theoretical ρ_mix, and porosity-corrected ρ_true.

This counterintuitive trend suggests that alumina promotes the formation of dense Bi–Ti–Al–O crystalline phases with reduced molar volumes relative to the nominal oxide mixture. The positive correlation between Al_2_O_3_ content and ρ_true therefore supports the hypothesis that Al plays a structural role in modifying the crystallization pathway toward denser titanate/orthotitanate phases. Overall, the comparative density analysis—combining measured ρ_a_, theoretical ρ_mix, and porosity-corrected ρ_true (Figure 16)—provides strong evidence for the following:

(i) The partial conversion of the parent glasses into high-density crystalline phases;
(ii) The structurally active incorporation of Al_2_O_3_;
(iii) Significant reduction in molar volume during crystallization; and
(iv) High densification (low porosity) in all samples, consistent with effective sintering and phase development.

## 4. Discussion

**Figure 17:**
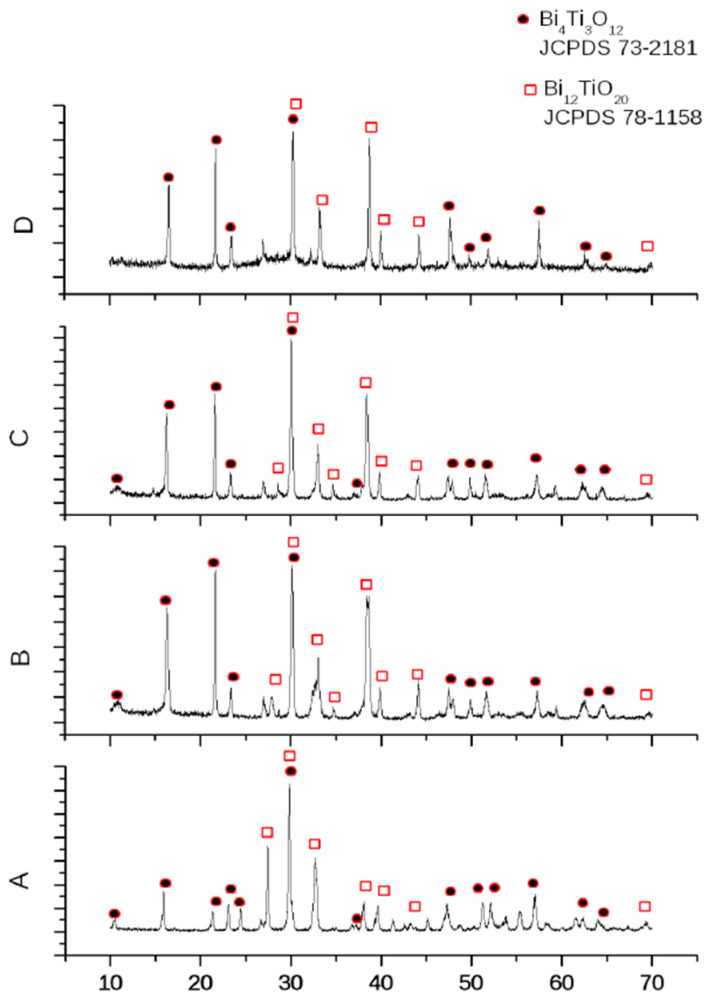
XRD patterns of samples: (A), (B), (C) and (D) [18].

As mentioned above, in our previous work [18], the compositions 45Bi_2_O_3_-50TiO_2_-5Nd_2_O_3_ (A), 36Bi_2_O_3_-60TiO_2_-4Nd_2_O_3_ (B), 33Bi_2_O_3_-60TiO_2_-3SiO_2_-4Nd_2_O_3_ (C), and 32Bi_2_O_3_-38TiO_2_-22SiO_2_-8Nd_2_O_3_ (D) were characterized with the crystallographic phases of the bismuth titanate Bi_4_Ti_3_O_12_ and Bi_12_TiO_20_, registered by XRD (Figure 17 [18]). These results are confirmed by FTIR (Figure 1) and Raman (Figure 2) the counterparts from which it is evident that 36Bi_2_O_3_-60TiO_2_-4Nd_2_O_3_ (B) additional absorption spectra of TiO_2_-based Ti-O bonds were noticed and in 33Bi_2_O_3_-60TiO_2_-3SiO_2_-4Nd_2_O_3_ (C) and 32Bi_2_O_3_-38TiO_2_-22SiO_2_-8Nd_2_O_3_ (D) SiO_2_/TiO_2_ compositions in the amorphous.

In sample 45Bi_2_O_3_-50TiO_2_-5Nd_2_O_3_ (A), absorption spectra of the Al-O-Al bonds, which entered the melt from the corundum crucible, were also observed. The probable combined influence of Al-O-Al and Ti-O bonds, as well as the presence of randomly arranged (untextured) bismuth–titanate phases, leads to a decrease in the real part of the dielectric constant and to the highest conductivity (compared to 36Bi_2_O_3_-60TiO_2_-4Nd_2_O_3_ (B), 33Bi_2_O_3_-60TiO_2_-3SiO_2_-4Nd_2_O_3_ (C), and 32Bi_2_O_3_-38TiO_2_-22SiO_2_-8Nd_2_O_3_ (D)), resulting from a minimal grain boundary effect. With the subsequent increase of TiO_2_ by 10 mol% in samples 36Bi_2_O_3_-60TiO_2_-4Nd_2_O_3_ (B) and 33Bi_2_O_3_-60TiO_2_-3SiO_2_-4Nd_2_O_3_ (C), these Ti-O bonds are suppressed, which leads to the formation of the Bi_4_Ti_3_O_12_ phase and explains their similar dielectric losses and conductivities. In the composition 33Bi_2_O_3_-60TiO_2_-3SiO_2_-4Nd_2_O_3_ (C), the introduction of 3 mol% SiO_2_ determines the appearance of distorted SiO_4_ polyhedra with broken Si–O–Si linkages, coexisting with Bi_4_Ti_3_O_12_ and Bi_12_TiO_20_ phases [18], which does not significantly change the dielectric losses but significantly increases the dielectric permittivity (real part). This effect is probably due to the formation of an amorphous boundary layer between the dielectric bismuth–titanate phases and the enhancement of the grain boundary effect. Further increase in the SiO_2_ content up to 22 mol% in sample 32Bi_2_O_3_-38TiO_2_-22SiO_2_-8Nd_2_O_3_ (D) leads to a reduction in TiO_2_ content and the appearance of a multicomponent amorphous matrix incorporating Al–O–Al, Si–O–Si, BiO_6_, Si–O–Ti, and SiO_4_ groups, which significantly reduce the dielectric losses and conductivity. The multicomponent amorphous matrix in this sample probably has a combined effect on the strong frequency dependence of its dielectric parameters.

### 4.1. Dielectric Properties

The dielectric response of the Nd_2_O_3_–SiO_2_–TiO_2_–Bi_2_O_3_ glass–ceramic samples was evaluated over the frequency range 1–106 Hz. The frequency dependence of the real permittivity ε′, dielectric loss ε″, AC conductivity σ_ac, and loss tangent tan δ for samples A–D is presented in Figure 7, Figure 8, Figure 9 and Figure 10. Clear correlations are observed between the dielectric behavior, the degree of densification, and the level of Al_2_O_3_ incorporation originating from the crucible.

All samples (Table 5) exhibit a characteristic decrease in ε′ with increasing frequency, consistent with Maxwell–Wagner [34] interfacial polarization and space-charge relaxation typically observed in heavy Bi_2_O_3_-containing glasses. At low frequencies, ε′ reaches values above 10^3^ for sample C and remains in the 4 × 10^2^–8 × 10^2^ range for samples A and B, while sample D exhibits significantly lower values (≈2 × 10^2^).

The magnitude of ε′ correlates strongly with the density hierarchy of the samples:

ε′(C) > ε′(B) > ε′(A) > ε′(D)

This alignment mirrors the measured true densities (ρ_true = 6.37 > 6.10 > 5.39 > 5.04 g·cm^−3^), indicating that higher densification enhances the polarizability by increasing the concentration of Bi–O, Ti–O and Bi–Ti–O units per unit volume. The exceptionally high ε′ values of sample C are further supported by its elevated Al_2_O_3_ incorporation (16.22 mol%), which promotes the formation of dense Bi–Ti–Al–O phases with high ionic polarizability.

The ε″ spectra reveal pronounced relaxation features in the 10^2^–10^3^ Hz region, particularly for samples C and B, indicative of dipolar and ionic relaxation processes. Sample C exhibits the highest dielectric loss across the entire frequency range, consistent with its highest ε′ and densest microstructure. In contrast, sample D, the least dense and most glassy composition, shows the lowest ε″ except for a small relaxation peak around 10^2^ Hz. The evolution of ε″ matches the trend in structural compactness and defect density: denser samples with crystalline Bi–Ti–O domains contain more polarizable units and oxygen vacancy sites capable of participating in hopping conduction. The incorporation of Al_2_O_3_ intensifies this effect by modifying the titanium–oxygen polyhedral network and increasing defect concentrations associated with charge compensation.

The AC conductivity increases with frequency for all samples, following Jonscher’s universal power law [38]:

σ_ac_(ω) = σ_dc_ + Aω_s_, 0 < s < 1

However, the sample ranking does not follow the monotonic density trend. Sample A exhibits the highest σ_ac_ at low frequencies, whereas sample C dominates at mid-to-high frequencies. Sample B shows intermediate behavior, while sample D presents the lowest σ_ac throughout the spectrum.

This indicates that σ_ac_ is governed primarily by defect chemistry rather than density alone, specifically the following:

- Oxygen vacancy concentration (originating from Bi^3+^ ↔ Ti^4+^ charge imbalance) [39,40],
- Degree of crystallization of conductive Bi–Ti–O phases,
- Presence of Al-induced defect states.

Sample A’s unexpectedly high σ_ac_ at low frequencies suggests a more open glassy network with enhanced ionic mobility, while sample C’s high σ_ac_ at high frequencies reflects strong hopping conduction through dense titanate crystallites.

The tan δ spectra (Figure 9) demonstrate pronounced dielectric loss for samples B and C, especially in the 10^3^–10^5^ Hz range, where tan δ exceeds values of 10–30. This behavior arises from the simultaneous occurrence of large ε′ and ε″ values in these compositions. The ranking tanδ(C) ≈ tanδ(B) > tanδ(A) ≫ tanδ(D) parallels the crystallization tendency and Al_2_O_3_ incorporation. Sample C, with the most significant Al uptake, shows the strongest loss response, highlighting the role of Al in promoting compact Bi–Ti–Al–O phases with enhanced dipolar relaxation. Sample D maintains the lowest tan δ (<1 across most frequencies), consistent with its low density, minimal crystallinity, and reduced defect concentration.

### 4.2. Structure–Property Relationship

The combined dielectric analysis reveals a consistent and physically meaningful correlation between dielectric response and the structural evolution of the samples:

Densification enhances ε′, especially in Al-enriched Bi–Ti–O phases.

Dielectric losses (ε″, tan δ) increase with the extent of crystallization and defect density (oxygen vacancies).

AC conductivity depends more on defect pathways and crystallite connectivity than on bulk density.

Al_2_O_3_ incorporation is a key structural driver, promoting dense Bi–Ti–Al–O crystallization and shifting dielectric properties upward in magnitude.

These trends confirm that dielectric behavior in this system is governed by the interplay between density, phase composition, oxygen vacancy concentration, and Al-induced structural modification.

### 4.3. Conduction and Polarization Mechanisms

The strong dispersion of ε′, ε″, σ_ac and tanδ with frequency (Figure 7, Figure 8, Figure 9, Figure 10, Figure 11, Figure 12, Figure 13 and Figure 14) indicates that the dielectric response of the Nd_2_O_3_–SiO_2_–TiO_2_–Bi_2_O_3_ glass–ceramics is governed by a combination of interfacial (Maxwell–Wagner) polarization and hopping conduction of localized charge carriers in the bulk.

### 4.4. Maxwell–Wagner Interfacial Polarization

The pronounced increase in ε′ at low frequencies, particularly for the dense and highly crystalline samples B and C, together with the large ε″ and tanδ in the same region, is typical of Maxwell–Wagner–Sillars (MWS) interfacial polarization. The microstructure consists of semiconducting or weakly conducting crystalline Bi–Ti–(Al)–O regions embedded in a more resistive glassy matrix and separated by grain boundaries. Under an applied AC field, charge carriers accumulate at these internal interfaces, giving rise to space-charge polarization and a large apparent permittivity at low frequencies. As the frequency increases, charge carriers cannot follow the rapidly alternating field and the interfacial contribution diminishes, causing ε′ to decrease and tan δ to pass through a maximum.

This picture is consistent with the density results: samples with higher ρ_true (B and C) contain a larger fraction of crystalline titanate phases and therefore a higher density of internal interfaces, strengthening the Maxwell–Wagner contribution. The more glassy sample D, with lower density and fewer interfaces, exhibits much weaker low-frequency dispersion.

### 4.5. AC Conductivity and Jonscher’s Power Law

The observed increase of σ_ac_ with frequency and the absence of a clear high-frequency plateau in the investigated range are characteristic of hopping conduction in disordered systems. In this framework, the exponent *s* reflects the degree of interaction and correlation between hopping charge carriers. For the present Bi_2_O_3_-rich glasses and glass–ceramics, the carriers are expected to be electrons or small polarons localized at defects such as Ti^3+^/Ti^4+^ centers and oxygen vacancies associated with Bi^3+^/Bi^5+^ and Al substitution. The relatively strong dispersion seen for samples B and C suggests *s* values significantly lower than unity, indicative of correlated hopping rather than simple Drude-type transport.

The normalized indices (Table 6) (Density Index = 1/W, Dielectric Quality Factor (High-f Q) = 1/tanδ_high, Stability Index = ε′_high/tanδ_high, Polarizability Index = ε′_low) help to compare all samples in a materials-selection style.

## 5. Conclusions

The influence of composition on the dielectric parameters of the samples within the studied concentration range can be summarized as follows:

1. Effect of Bi_2_O_3_/TiO_2_ ratio:

When Bi_2_O_3_ and TiO_2_ are present in comparable amounts, more than one bismuth–titanate phase can form, with the lower-temperature phase—Bi_12_TiO_20_ in sample 45Bi_2_O_3_–50TiO_2_–5Nd_2_O_3_ (A)—appearing first. Under these conditions, a tendency toward the formation of Ti–O bonds is observed. In cases where the crystal phases grow in a disordered manner, this may lead to increased electrical conductivity.

2. Effect of limited SiO_2_ addition:

The incorporation of small amounts of SiO_2_ leads to an increase in dielectric permittivity due to the formation of Si–O–Si boundary chains between the crystalline phases.

3. Effect of larger SiO_2_ content:

Increasing the SiO_2_ concentration—particularly to levels comparable to that of TiO_2_, as in sample 32Bi_2_O_3_–38TiO_2_–22SiO_2_–8Nd_2_O_3_ (D)—promotes the formation of a multicomponent amorphous matrix consisting of Si–O–Si, BiO_6_, Si–O–Ti, and SiO_4_ structural groups. This results in a pronounced reduction in dielectric losses and electrical conductivity. According to the results of this study, a higher proportion of amorphous matrix (sample D) increases the volume resistivity. Although the grain-boundary contribution remains significant and the total resistance is the lowest among the samples, sample D also exhibits a slight decrease in both the real part of the dielectric constant and the dielectric losses.

In summary, the synthesis of bulk materials by the melt-quenching method, combined with the introduction of amorphous-forming oxides such as SiO_2_ (and Al_2_O_3_), provides a promising approach for reducing dielectric losses and electrical conductivity. Embedding the crystalline bismuth–titanate phases within an amorphous matrix promotes this effect. Furthermore, the development of a preferred orientation of the bismuth–titanate phases is advantageous for enhancing the real part of the dielectric permittivity.

The structure–property relationships of the Nd_2_O_3_–SiO_2_–TiO_2_–Bi_2_O_3_ glass–ceramic system were systematically established by correlating density, Al_2_O_3_ incorporation, degree of crystallization, and dielectric behavior. The measured apparent densities (4.99–6.12 g·cm^−3^) and porosity-corrected true densities (5.04–6.37 g·cm^−3^) clearly reflected the extent of structural compaction and phase development across the series. Samples B and C, which exhibited both the highest densities and the greatest Al_2_O_3_ uptake from the crucible (2.98 and 16.22 mol%, respectively), showed the strongest tendency toward crystallization into Bi–Ti–Al–O titanate phases with reduced molar volume.

These structural differences directly governed the dielectric response. Densely crystallized samples displayed the highest ε′ and ε″ values, consistent with enhanced polarizability and a larger population of localized charge carriers. Sample C exhibited the highest permittivity and loss tangent over most of the frequency range, reflecting the combined influence of heavy Bi-containing phases and Al-modified Ti–O polyhedra. By contrast, sample D, the least dense and most glassy composition, showed markedly lower ε′, ε″, and σ_ac, indicating limited polarizability and reduced hopping conduction.

The frequency dependence of σ_ac_ followed Jonscher’s power law, while the combined dispersion of ε′, ε″, and tan δ revealed a two-stage dielectric mechanism: (i) Maxwell–Wagner interfacial polarization at low frequencies due to semiconducting crystalline domains embedded in a resistive glass matrix, and (ii) correlated barrier hopping (CBH) of localized carriers at higher frequencies, associated with oxygen-vacancy-related states in the Bi–Ti–(Al)–O network.

Overall, the results demonstrate that densification, Al_2_O_3_ incorporation, and titanate crystallization act synergistically to enhance dielectric polarization and hopping transport, while more glassy and less compact structures suppress these effects. These findings highlight the critical role of composition–structure interactions in tuning the electrical performance of heavy-oxide glass–ceramic systems and provide a solid framework for optimizing both dielectric and conductive properties through controlled crystallization and compositional engineering.

Considering the phase composition (Bi_4_Ti_3_O_12_ and Bi_12_TiO_20_) identified in our previous studies, as well as the observed tendency for orientation along the crystallographic c-axis, several directions for future research can be outlined. These include a broader investigation of compositions within the Bi–Ti–Si–Nd system to define the domain of glass formation, as well as an evaluation of the influence of aluminum inclusions originating from melting in corundum crucibles.

## Figures and Tables

**Table 1:** Starting materials, phase composition, and melting conditions of selected samples in the Bi_2_O_3_–TiO_2_–SiO_2_–Nd_2_O_3_ system [18].

Name of the Sample	Composition, mol %				T_m_, °C	Heating Time, min	Phases, According to XRD
	Bi_2_O_3_	TiO_2_	SiO_2_	Nd_2_O_3_			
A	45	50	0	5	1100	15	Bi_4_Ti_3_O_12_, Bi_12_TiO_20_
B	36	60	0	4	1450	20	Bi_4_Ti_3_O_12_ (tex), Bi_12_TiO_20_
C	33	60	3	4	1450	20	Bi_4_Ti_3_O_12_ (tex), Bi_12_TiO_20_
D	32	38	22	8	1450	20	Bi_4_Ti_3_O_12_ (tex), Bi_12_TiO_20_

**Table 2:** The specific sizes of the samples.

Samples	Thickness, mm	Diameter, mm
A	2.32	4.1
B	2.1	5.77
C	4.93	4.27
D	3.11	4.6

**Table 3:** Water absorption of the selected samples.

Sample	Sintering T (°C)	Water Absorption W (%)	Microstructural Implication
A	1100	0.41	moderately dense
B	1450	0.68	least dense; most residual porosity
C	1450	0.62	moderate porosity; thin glassy phase
D	1450	0.18	highest density; strong liquid-phase sintering

**Table 4:** Experimental, theoretical and porosity-corrected values.

Sample	Al_2_O_3_ (mol%)	ρ_a_ (g/cm^3^)	P_a_ (%)	ρ_Mix (g/cm^3^)	ρ_True (g/cm^3^)
A	1.30	5.28	2.14	5.69	5.39
B	2.98	5.85	4.00	5.32	6.10
C	16.22	6.12	3.81	5.07	6.37
D	7.84	4.99	0.89	4.32	5.04

**Table 5:** Dielectric performance summary (based on Figure 7, Figure 8, Figure 9 and Figure 10).

Sample	ε′ (Low f, ~1 Hz)	ε′ (High f, 10^5^–10^6^ Hz)	tanδ (Low f)	tanδ (Peak)	tanδ (High f)
A	600	300–350	2–5	10	15–20
B	200	150–180	3–6	10–12	15–30
C	1000	400–500	2–5	10	10–15
D	300	350–400	0.2–0.4	0.6	0.1–0.3

**Table 6:** The normalized indices DI, Q_high_, SI, PI.

Sample	Density Index (1/W)	Q_High (~1/tanδ)	Stability Index ε′/tanδ	Polarizability Index ε′
A	2.44	~0.07	~20	600
B	1.47	~0.04	~10	200
C	1.61	~0.07–0.1	~35–40	1000
D	5.55	3–10	~1500–3000	300

Best sample in each category are the following: highest density: D; highest stability: D; highest Q: D; highest polarizability: C.

## Data Availability

The original contributions presented in this study are included in the article. Further inquiries can be directed to the corresponding author.
